# Morphometric Analysis of Anatomy of Anterior Cruciate Ligament of Knee and its Attachments - a Cadaveric Study in Indian Population

**DOI:** 10.5704/MOJ.2111.002

**Published:** 2021-11

**Authors:** S Mishra, A Mylarappa, D Satapathy, S Samal

**Affiliations:** Department of Orthopaedics, Siksha O Anusandhan University Institute of Medical Sciences and SUM Hospital, Bhubaneswar, India

**Keywords:** anterior cruciate ligament, morphometric, gender, posterolateral, anteromedial

## Abstract

**Introduction:**

The Anterior Cruciate Ligament tends to stabilise the knee in various range of extension and flexion. Precise study of anatomy, attachments and position of bundles is important for successful ACL reconstruction. In our study, we attempt to assess general anatomy of ACL, determine and compare its morphometric data pertaining to length and width and its tibio-femoral foot prints in different gender and secondarily determine changes in the same during ACL dynamics witnessed during knee flexion changes.

**Materials and methods:**

A total of 19 knees from 10 cadavers were used in the research with mean age of 61±7 years. After dissecting the skin, muscles, patellar and articular capsule were removed and bundle attachments were studied. Thereafter the relative length, width and stiffness of ACL bundles at 0, 90, 140 (maximum) angles of knee flexion were measured along with maximum horizontal and vertical bundle footprints at tibio-femoral attachments were recorded.

**Results:**

Mean length and width of insertion of anteromedial (AM) bundle on the tibial surface was 8.8mm and 9.0mm in males and 8.1mm and 8.8mm in females. Furthermore, that of PL bundle was 9.1mm and 7.8mm in males and 8.9mm and 7.1mm in females.

**Conclusion:**

The anteromedial (AM) bundle and posterolateral (PL) bundle of ACL were found to be most relaxed at full extension and were most taut at maximum flexion of 140°. AM bundle underwent greater stretching and change of length in comparison to the PL bundle, indicating that it is comparatively a more dominant bundle.

## Introduction

Knee is a complex synovial joint. The cruciate ligaments are the main stabiliser of knee joints in various degrees of flexion-extension. The ACL is a tough band of fibrous tissue which runs diagonally from posteromedial aspect of lateral femoral condyle to the anterior intercondylar areas of the tibial plateau. ACL prevents the anterior translation of tibia on the femur, helps in rotational movements and also helps in controlling the back and forth movements of the lower leg. Standard anatomical textbooks describe the average length and width of an adult ACL to be around 40mm and 12mm, respectively and state that it is made up of two or possibly three functional bundles namely anteromedial, intermediate, and posterolateral^1^. But most of the published research works have revealed that ACL consists of two bundles namely anteromedial (AM) bundle and posterolateral (PL) bundle according to their femoral attachments^2^. Nevertheless, ambiguity in this regard persists.

In recent years there has been a worldwide increase in road traffic accidents and increased participation in sporting activities which in turn has led to an increase in the incidence of anterior cruciate ligament (ACL) injury^3^. As a result ACL reconstruction has become one of the most common surgical procedures performed by orthopaedic surgeons^2^ all over the world including India. In India alone ACL injuries account for 60% of all acute knee injury cases. This is expected to increase in coming years, particularly in females who have a higher risk of developing ACL injury^3,4^. Symptomatic ACL injuries are managed with surgical reconstruction on most occasions. The trend is towards reconstruction techniques that more closely restore native ACL anatomy^5^. In anatomic ACL reconstruction the graft is placed in the anatomic position, with tunnels drilled directly through the original attachment site, using either the single or double bundle technique^6^. No additional advantage of the double bundle reconstruction over the single bundle reconstruction has been proven clinically^7^ especially when an anatomic reconstruction is performed^6^. The tibial insertion size, viewed arthroscopically, plays a role in determining the technique to be selected^8^ as a tibial attachment size of less than 14mm is too small to accommodate double bundle reconstruction^9^. Thus determination of exact anatomical location of the tibial and femoral attachments of the ACL, the degree of obliquity of fibres and the exact nature of AM and PL bundles are of paramount importance before drilling of the tunnels for placements of the ACL grafts.

It is also known that ACL is made up of collagen fibrils. The amount of collagen fibril in a population changes during maturation and aging which increases the level of stress on the ligament^10^. Thus determination of fibril diameters of ACL is very important during its reconstruction surgeries. If left untreated, most of these injured knees progress to secondary osteoarthritis with significant damage to menisci and articular cartilage which is a long term morbidity, sometimes requiring replacement arthroplasty. Hence, morphometric analysis of correct anatomy of ACL, its associated bundles, thickness of its fibres, and relative positions during motion is highly indispensable to plan a proper surgical reconstruction.

Our study attempts to contribute further to the database on ACL anatomy in the context of the Indian population and its gender variation. The study aims to determine and check relationships of these measurement data pertaining to length and width of ACL and its tibio-femoral foot prints in the right and left knees of an individual and trace out variation among male and female. It further aims to determine changes in these measurements while ACL dynamics change during knee flexion. Such information could be useful in achieving anatomical ACL reconstruction and thus individualise the procedure which can result in better surgical outcomes and reduce failure rates where wrong anatomical data might have resulted in wrong choice of tunnel placement and incorrect type of bundle replacement which can vary widely from the western literature and other neighbouring Asian countries.

## Materials and Methods

A total of 10 cadavers were randomly selected for the study from all the available cadavers in the Anatomy Department of Institute of Medical Sciences and SUM Hospital under Siksha O Anusandhan University. These included six male and four female cadavers. All the cadavers were embalmed with 10% formalin mixture. Since most of the cadavers were unclaimed no consent from their families were necessary. The average age was 61±7years (range 45-75 years) as per the municipal records. The height and weight of the cadavers were noted (Table I). None of the knees had any significant visible injuries around the knee. One knee which was partially amputated was not included in the study. Ten left and nine right sided knees were thus obtained.

**Table I:** Demographic Parameters

Cadavers	Age	Sex	No of knees	Height (cm)	Weight (kg)
1	55	male	2	178	66
2	70	male	2	168	60
3	64	female	2	156	58
4	45	female	2	160	70
5	68	male	2	184	75
6	75	male	2	182	84
7	62	male	2	175	64
8	49	female	1	162	58
9	57	male	2	172	68
10	72	female	2	158	62
Average	61.7			169.5	66.5

**Fig. 1:**
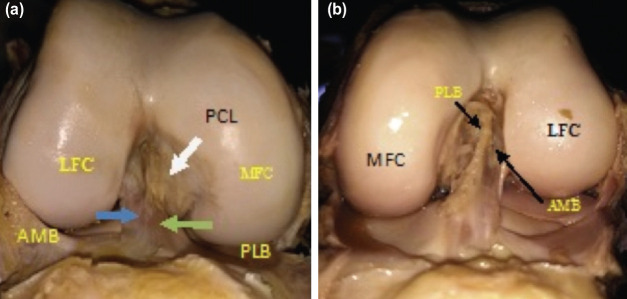
(a) AM and PL bundles in right knee (male). (b) AM and PL bundles of ACL in left knee (male).

**Fig. 2:**
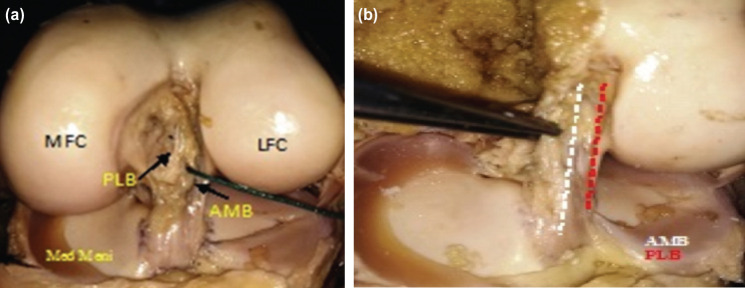
(a) AM bundle ACL demonstration. (b) PL bundle demonstration.

**Fig. 3:**
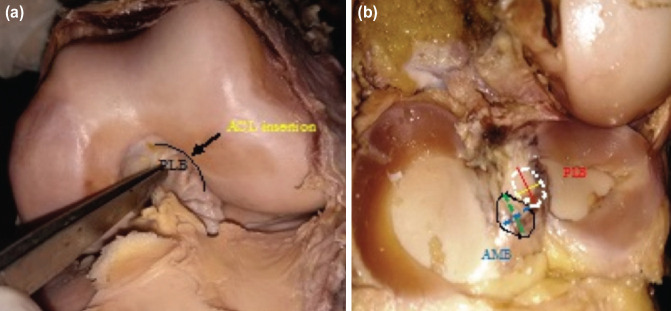
(a) Femoral attachment of ACL in a female. (b) Tibial foot print.

**Fig. 4:**
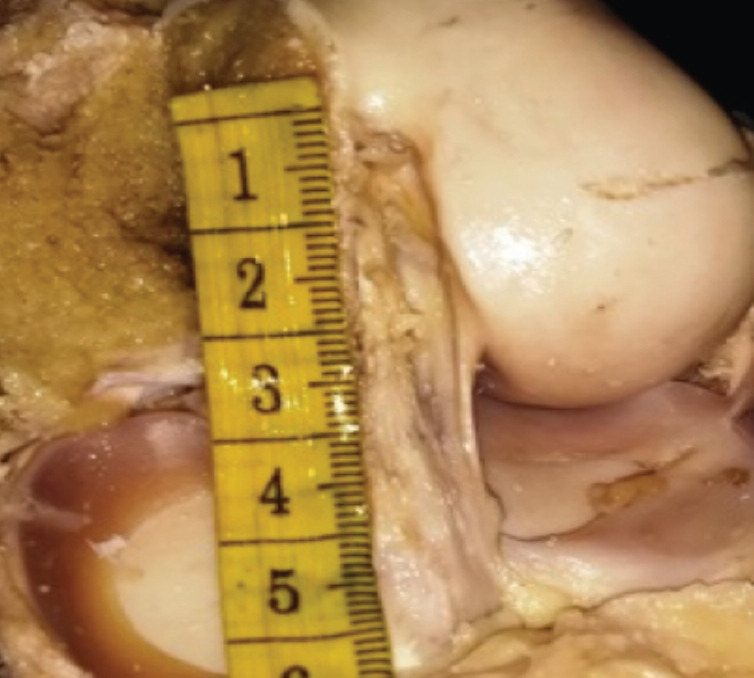
Demonstration of ACL length using flexible ruler (for demonstration).

**Fig. 5:**
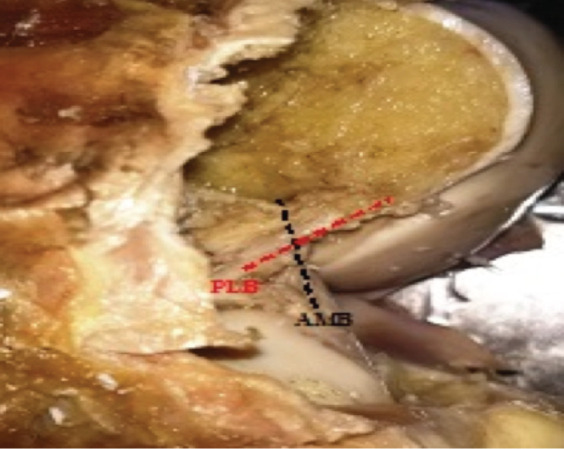
Twisting of bundles at 90° knee flexion.

The knees were cleared of all surrounding tissues except the anterior cruciate ligament (ACL) and posterior cruciate ligament (PCL), the meniscus, the collaterals, the surrounding soft tissues and vessels. Fat were removed to make the visualisation of the ACL clearer. The intercondylar portion of the femur was sectioned between the insertions of the ACL and PCL taking care to preserve the attachment sites and the ACL attachment sites were thus exposed. The bundles of the ligament were identified (Fig. 1 and 2). The Length of ACL was measured using a flexible ruler (Fig. 3). The two bundles were marked by wrapping a string. The change in length in the ACL bundles were noted at 0°, 90° and maximum knee flexion 140° (Fig. 4). After that the PCL ligament was excised to expose the femoral attachments of the bundles and their attachment sites were outlined with a marker after which the bundles were sectioned from the bone. The maximum anterior-posterior and horizontal diameters of both the tibial and femoral attachment sites and the footprints areas were measured using a digital Vernier caliper (aero space accuracy of 0.02mm) (Fig. 5). Thereafter, digital photographs were taken.

Two observers took measurements of each parameter separately and recorded their averages. The length and width of the ACL tibial insertion was measured as the maximum distance between the anterior and the posterior edge of the tibial insertion and the width was measured at its maximum width of insertion on tibial condyle. The length and the width of the insertion on the femur were measured in maximal flexion of the knee joint. The length of the femoral insertion was measured from the proximal most point of insertion to the distal most point of ACL insertion. The width of the femoral insertion was measured as maximum vertical distance from line of femoral insertion till the ending of insertion just below the articular surface of femur. The length of the AM bundle of the ACL was measured from the anterior edge of the tibial insertion to the medial half of femoral insertion of ACL on femoral condyle in maximum knee flexion. The length of PL Bundle was measured from the posterior edge of tibial insertion to the lateral half of femoral insertion.

All measurements were carried out using digital Vernier callipers, flexible tape and photographs were obtained of the same. All measurements recorded were tabulated and analysed. Data was presented as means ± standard deviation. To test for the differences between male and female gender and between right and left sides of the body, an analysis of variance (ANOVA) was conducted. With the P value set at <0.05=significant, <0.01=highly significant, >0.05=not significant.

## Results

Since most of cadavers were unclaimed dead bodies received by the hospital authorities from civic bodies, prior history of their jobs, activities, health and lifestyle were not available for the purpose of the study.

In our study mean length and width of AM bundle was observed to be 40.17mm and 7.83mm, respectively while that of PL bundle was found to be 28.42mm and 5.5mm, respectively. There was significant difference (p< 0.05) in the length and width of AM and PL in male in comparison to female. In male the mean length and width of AM bundle was 40.9mm and 8.0mm while that of female was 36.5mm and 7.0mm, respectively. Similarly length and width of PL bundle in male was 29.0mm and 5.6mm and 27.2mm and 5.0mm in female, respectively. There was no significant difference in lengths of AM and PL bundles in the right and left knees (p>0.05). All the measurements were done at maximum flexion of knee (Table II).

**Table II:** ACL length (maximum length in full knee flexion)

	Anterio Medial bundle						Postero Lateral bundle					
	Mean length (mm)	SD	P Value	Mean width (mm)	SD	P Value	Mean length PL bundle (mm)	SD	P Value	Mean width (mm)	SD	P Value
Mean	40.17	2.41		7.83	1.03		28.42	1.44		5.5	0.56	
Male	40.9	1.85	0.025	8.0	1.05		29.0	1.25	0.046	5.6	0.52	0.031
Female	36.5	0.71		7.0	0		27.2	1.41		5.0	0	
RT Knee	40	2.28	0.621	8.2	0.89		28.67	1.51	0.327	5.5	0.54	0.28
LT Knee	40.33	2.73		7.9	1.21		28.17	1.47		5.5	0.52	

Parameters of tibial attachment site including mean length and width of insertion of AM bundle on the tibial surface was 8.8mm, 9.0mm in male and 8.1mm, 8.8mm in female. The parameters of PL bundle were 9.1 mm, 7.8mm in male and 8.9mm, 7.1mm in females. The AM bundle had a larger coverage area of the total ACL insertion site on the tibial foot plate in comparison to PL bundle. AM bundle occupied anteromedial portion of the tibial plateau and was just medial to tibial spine with a semi-lunar cross sectional area. The PL bundle had a more oval cross section and was located just lateral to tibial spine. No significant differences were found in left and right sides (Table III).

**Table III:** Tibial foot print measurements

	Anterio Medial bundle						Postero Lateral bundle					
	Mean length (mm)	SD	P Value	Mean width (mm)	SD	P Value	Mean length PL bundle (mm)	SD	P Value	Mean width (mm)	SD	P Value
Mean	8.4	2.02		8.9	0.64		8.0	1.77		7.6	1.42	
Male	8.8	0.17	0.041	9.0	1.05	0.037	8.1	0.34	0.061	7.8	0.52	0.035
Female	8.1	0.7		8.8	0		7.9	1.41		7.1	0.2	
RT Knee	8.7	0.21	0.164	8.9	0.89	0.211	8.2	1.50	0.082	7.9	0.55	0.052
LT Knee	8.6	1.06		8.8	0.81		8	0.47		7.2	0.55	

Parameters of Femoral attachment site including mean length and width of insertion of AM bundle on the femoral surface was 8.8mm, 7.1mm in male and 7.8mm, 7.0mm in female. The parameters of PL bundle were 7.4 mm, 6.8mm in male and 7.1mm, 6.4mm in female. The AM bundle had oval cross sectional area and occupied the posterior-inferior part of the inner surface of the lateral condyle while the PL bundle had a semilunar cross sectional area and was located anterior to the AM bundle attachment over lateral femoral condyle. No significant differences were found in left and right sides (Table IV).

**Table IV:** Femoral attachment measurement

	Anterio Medial bundle						Postero Lateral bundle					
	Mean length (mm)	SD	P Value	Mean width (mm)	SD	P Value	Mean length PL bundle (mm)	SD	P Value	Mean width (mm)	SD	P Value
Mean	8.7	0.56		7.1	2.13		7.3	1.62		6.6	0.72	
Male	8.8	1.07	0.019	7.1	0.44	0.052	7.4	0.04	0.038	6.8	1.02	0.047
Female	7.8	0.24		7.0	0		7.1	0.41		6.4	0.26	
RT Knee	8.4	1.01	0.054	7.16	0.74	0.121	7.3	1.54	0.081	6.7	0.75	0.073
LT Knee	8.2	2.02		7.2	0.02		7.2	0.07		6.5	1.06	

The anatomical position change of two bundles was evaluated from full extension to full knee flexion which was about 140° in almost all cases. AM and PL were parallel in the sagittal plane with full extended knee and the AM bundle was located in the anterior proximal aspect of the PL bundle, however, the bundles twisted around each other with increasing degrees of knee flexion and femoral insertion site of AM and PL bundles were displaced to posterior and anterior, respectively.

Stretching of the AM and PL bundle were evaluated by measuring their lengths at 0°, 90° and full flexion (140°). There was significant alteration in length of both bundles at various degrees of flexion. Both AM and PL bundles were taut in flexion from 30° onwards. While the length of AM bundle changed from 33.4mm in extension to 40.17 in full flexion, the length of PL bundle changed from 24.2mm in extension to 28.42mm in full flexion. Similar patterns were seen in both sexes and in right and left knee (Table V). The AM bundle underwent more stretching and change of length in comparison to the PL bundle indicating that it is the more dominant bundle in the ACL ligaments and more attention needs to be given for its proper reconstruction especially in a double bundle ACL reconstruction.

**Table V:** Variation in ACL length in various degrees of flexion and extension

	Anterio Medial bundle						Postero Lateral bundle					
	Mean length (mm) in full flexion (130°-140°)	SD	Mean length in 90° flexion	SD	Mean length (mm) in full extension	SD	Mean length (mm) in full flexion (130°-140°)	SD	Mean length in 90° flexion	SD	Mean length (mm) in full extension	SD
Mean	40.17	2.41	39.6	2.36	33.4	1.43	28.42	1.44	26.9	2.54	24.2	0.67
Male	40.9	1.85	38.8	1.07	34.6	2.07	29.0	1.25	27.4	1.74	25.6	1.22
Female	36.5	0.71	35.4	2.51	30.2	1.6	27.2	1.41	25.8	0.44	22.7	2.13
RT Knee	40.0	2.28	38.9	0.82	33.8	2.31	28.67	1.51	27.2	2.01	23.5	1.56
LT Knee	40.33	2.73	39.2	1.58	35.4	0.55	28.17	1.47	26.8	1.79	24.1	0.27

## Discussion

In all the cadavers we found that ACL consisted of two separate bundles arranged as one anteromedial bundle and one posterolateral bundle. This was consistent with various literatures and works of Colombet et al and Steckel *et al* who also reported a double bundle ACL^11,12^ but was contradictory to reports of Ondensten et al who found a single bundle^13^ and Hollis *et al* who reported three bundles in ACL anatomy^14^. Our finding lays more importance on double bundle reconstruction in ACL repair which is a more anatomic and stronger construction than a single bundle reconstruction.

One of the important factors predicting success in Double Bundle ACL reconstruction technique is graft tunnel placement, which requires accurate description of the bundle attachment on femur and tibia surface^15^. In our study, the femoral attachment of AM bundle was in the posterior-inferior part of the inner surface of the lateral condyle and PL bundle was in its anterior aspect- which was slightly different from the reports of Muneta *et al* and Yasuda *et al* who reported a purely posterior attachment on the inner surface of the lateral femoral condyle^16,17^. On the tibial surface the tibial spine separated the attachment of AM and PL bundles. This study also corroborates the finding of tibial foot print measurement of ACL insertion with mean length and width of AM bundle being 8.4mm and 8.9mm while that of PL bundle being 8.0mm and 7.6mm which was similar to arthroscopic observations of who reported AM at 9.1mm and 9.2mm while PL bundle was 7.4mm and 7mm, respectively8. Finding of PL bundle were also similar to findings of Ferretti *et al* who reported measurements of 8.7mm and 7.9 mm for PL bundle^18^.

It was observed that the AM and PL were parallel in the sagittal plane with full extended knee and the AM bundle was located in the anterior aspect of the PL bundle. However, the bundles twisted around each other with increasing degrees of knee flexion toward 90° and femoral insertion site of AM and PL bundles were displaced to posterior and anterior, respectively. This was consistent with Hollis *et al*, Steckel *et al* and Duthon et al, who all reported the parallel and twisted orientation in full extension and 90° flexion, respectively^14,15,19^. It was observed that the femoral origin of the PL bundle is posterior and inferior to the AM bundle when knee is extended, while it is anterior to the origin of AM bundle in full flexion on the lateral femoral condyle^16^. Considering this position is essential especially in arthroscopic surgery in which the knee is flexed.

During the course of flexion the AM and PL bundles were significantly stretched which produced change in its length. Both the AM and PL had significant length change in extension (mean length 33.4mm and 24.2mm in extension) which changed to 40.14mm and 28.42mm in full flexion of 140°. This was consistent with findings of Cayir *et al* who reported significant length changes in both bundles with increasing flexion^20^. This is important in determining appropriate length of ACL graft keeping in mind the amount of stretching that it may be subjected to on full flexion as arthroscopic procedures are carried out at 90° knee flexion^21^. Our study had a few limitations. The study was limited by the availability of fewer number of cadavers. The mean age of cadavers was on the higher side of 61 years. Some of the cadavers used in the study had been preserved for more than one year, which might have been affected by tissue and ligamentous changes including easy friability and dehydration, making the ligaments tougher and harder. Variations in morphometric parameters could be because of manual observer variation and the different methods used for length measurement. Nonetheless our findings were consistent with some of the already published literature in this regard. More studies may be needed in the future, with a larger sample size for demonstrating anatomic variations in human population including gender, age and race variations.

## Conclusion

Our study demonstrated morphometric parameter of ACL and its variation among male and female sexes and between right and left knees in the Indian population. This can help surgeons make critical decisions concerning graft size, preference for double bundle reconstruction and appropriate position of tunnels in the tibial and femoral counterparts in arthroscopic ACL reconstruction procedures. The study also demonstrated the change in length of ACL bundles during various degrees of flexion. The study shall be helpful in decision making pertaining to the appropriate length of graft, so as to prevent a reconstruction which is very rigid or lax during various ranges of motion.

Furthermore, the study can help the average Indian Orthopaedician in better understanding of knee anatomy in native population, which is significantly different from Western and other Asian countries. This will result in more successful outcomes and decreased failure in decision making in the future. The limitations of the study can be further addressed by taking a larger sample size and using radiological parameters like MRI into account.
